# Effect of plasma-activated water against *E. coli* and *S. aureus*: Influence of organic matter and impact on skin cell viability

**DOI:** 10.1007/s00253-025-13635-7

**Published:** 2025-11-10

**Authors:** Yelyzaveta Moiseienko, Hafiz Muhammad Shahbaz, Saliha Saad, Matthew B. Avison, Alexandros Ch. Stratakos

**Affiliations:** 1 Centre for Research in Sustainable Agri-food & Environment, School of Applied Sciences, University of the West of England, Bristol, UK; 2 Department of Nutrition and Health, United Arab Emirates University, College of Medicine and Health Sciences, Al Ain, United Arab Emirates; 3 School of Cellular & Molecular Medicine, Biomedical Sciences Building, University of Bristol, Bristol, UK

**Keywords:** Plasma-activated water, Biofilm inactivation, Organic matter interference, *Escherichia coli* O157:H7, *Staphylococcus aureus*, Cytotoxicity

## Abstract

**Abstract:**

The study evaluated the antimicrobial efficacy of plasma-activated water (PAW), generated using a plasma bubble reactor, against *Escherichia coli* O157:H7 and *Staphylococcus aureus* in both planktonic and biofilm states. The physicochemical properties of PAW, including pH, electrical conductivity, and reactive oxygen and nitrogen species concentrations, were analysed immediately after production and after 24 and 48 h of storage at 4 °C. Additionally, the impact of organic load on PAW's antibacterial activity and its cytotoxic effects on human keratinocytes were investigated. To assess its stability, PAW’s antimicrobial activity after storage was also evaluated in the presence and absence of organic matter. PAW treatment resulted in a significant reduction in bacterial counts, achieving inactivation below the detection limit (1 log CFU/mL) within 20 min for both planktonic and biofilm states. However, the presence of organic matter significantly impaired PAW’s antibacterial efficacy, with higher organic loads leading to significantly diminished bacterial inactivation. PAW stored for 24 h maintained strong antimicrobial activity, which declined after 48 h; the presence of organic matter further reduced its efficacy across all time points. Importantly, PAW’s exposure did not induce cytotoxic effects on human keratinocytes at treatment durations of up to 30 min. These findings highlight the potential of PAW as a sustainable disinfection strategy, demonstrating robust antimicrobial activity against Gram-negative and Gram-positive foodborne pathogens while maintaining biocompatibility. Further research is required to enhance PAW’s efficacy in complex environments with organic contamination to enhance its practical applications in agri-food settings.

**Key points:**

*• *PAW effectively inactivated E. coli and S. aureus in planktonic and biofilm states.

*• *PAW antimicrobial activity is reduced in the presence of organic matter.

*• *PAW showed minimal cytotoxic effects on human keratinocytes.

**Graphical Abstract:**

**Figure:**
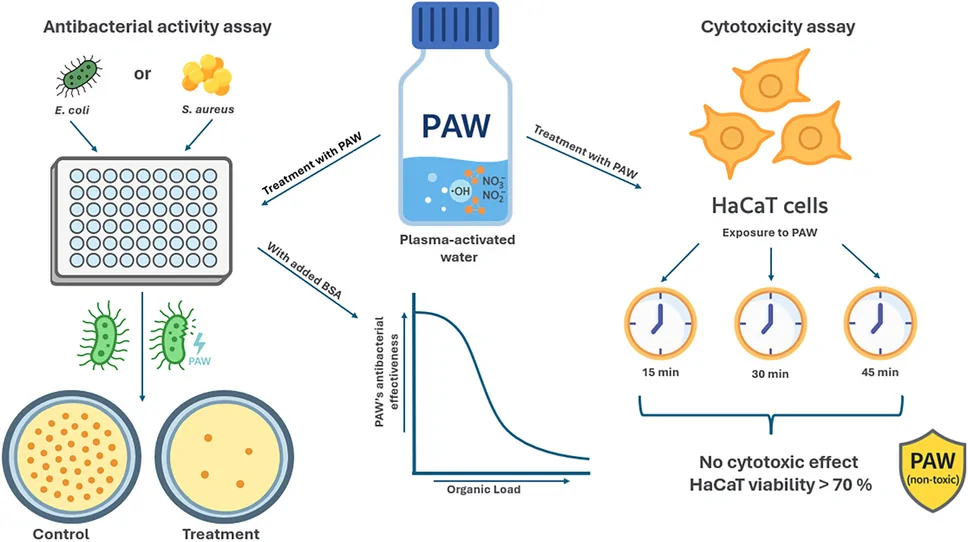

## Introduction

Foodborne illnesses remain a significant public health concern worldwide, with microbial contamination occurring at various stages of the food production chain, including farming, processing, packaging, and transportation (Tropea 2022). According to the World Health Organization (WHO), foodborne pathogens are among the leading causes of illness and mortality, contributing to substantial economic losses and food waste (World Health Organization 2025). Contaminated food can lead to severe infections, often presenting as nausea, vomiting, diarrhoea, and fever, with some cases progressing to life-threatening complications such as haemolytic uremic syndrome (HUS) or septicaemia (Kemper 2012; Lianou et al. 2017; U.S. Food and Drug Administration 2023).

The majority of foodborne infectious diseases are caused by pathogenic bacteria, such as *Escherichia coli, Staphylococcus aureus*, *Salmonella Typhimurium* and *Listeria monocytogenes* (Lianou et al. 2017; Ansari et al. 2022). In 2011, a total of 3,842 cases of enteroaggregative haemorrhagic *E. coli* O104:H4 infection, including 53 fatalities, were reported in Germany. Among these cases, more than 800 adults and over 90 children developed HUS caused by Shiga toxin-producing *E. coli* O104:H4, resulting in the largest paediatric HUS epidemic on record (Beutin and Martin 2012; Kemper 2012). Later, in 2015, 16 European Union member states reported a total of 453 foodborne outbreaks caused by staphylococcal toxins (Ercoli et al. 2017). Another case of massive food poisoning was reported in Vietnam in 2018, with a total of 352 children hospitalised. *S. aureus* and its enterotoxins were found in multiple food products consumed by the children at lunch (Le et al. 2021). *S. aureus*, which is responsible for the production of dangerous toxins, is also normally part of the human skin microbiota (Ercoli et al. 2017; Zhao et al. 2023b).

A recent outbreak of Shiga toxin-producing *E. coli* O145 occurred in the United Kingdom between May and June 2024, resulting in 275 confirmed cases and two reported deaths within 28 days of infection confirmation. Epidemiological and food chain investigations identified contaminated lettuce in pre-packaged sandwiches as the primary source of the outbreak (UK Health Security Agency 2024). In October 2024, a multi-state outbreak of *E. coli* O157:H7 occurred in the United States, with 104 reported infections, 34 hospitalizations, and one death. Traceback analysis identified fresh slivered onions, served at McDonald’s restaurants, as the likely source of the contamination (U.S. Food and Drug Administration 2024) A recent outbreak of *S. aureus* food poisoning occurred in 2024 in China, affecting 64 individuals and resulting in symptoms such as diarrhea, vomiting, abdominal pain, and nausea. The outbreak was linked to unsafe kitchen practices and poor food handling hygiene (Zheng et al. 2025).Furthermore, these pathogens can form biofilms on food and food contact surfaces, which enhances their persistence and increases the risk of contamination (Zhao et al. 2023a). Current food decontamination strategies rely on chemical agents (e.g., chlorine, ethanol, hydrogen peroxide, ozone) and physical methods (e.g., heat, radiation, ultrasound, UV treatment) (Mendoza et al. 2022; Rothwell et al. 2023). However, there is a growing shift towards more sustainable and safer antimicrobial strategies, driven by concerns over chemical residues, environmental impact, and consumer health.

One approach that has gained significant interest across various fields due to its unique properties and wide range of applications is novel plasma-activated water (PAW) technology (Liu et al. 2020; Zhou et al. 2020; Rothwell et al. 2023). PAW is produced by treating water with cold atmospheric plasma (CAP), which alters its physicochemical properties, including pH, oxidation–reduction potential (ORP), and electrical conductivity (EC) (Tan and Karwe 2021; Oliveira et al. 2022; Han et al. 2023). Contact of cold atmospheric plasma with water induces the generation of initial reactive species such as hydroxyl radicals (*·OH*), atomic oxygen (*O*), atomic nitrogen (*N*), superoxide (*O₂⁻*), and nitric oxide (*NO*). These species, in turn, contribute to the production of secondary reactive species, including hydrogen peroxide (*H₂O₂*), ozone (*O₃*), nitrates (*NO₃⁻*), nitrites (*NO₂⁻*), peroxynitrite (*ONOO⁻*), nitric oxide (*NO*_*x*_), and various ions (Gao et al. 2022). It is widely believed that alongside acidic pH, reactive oxygen and nitrogen species (RONS) are primarily responsible for PAW’s antimicrobial activity (Qian et al. 2019; Wong et al. 2023). Researchers have noted that the physicochemical properties of PAW and RONS concentrations depend on the conditions under which the activated water is generated, including the type of plasma source, treatment duration, gas composition, humidity, and other critical factors (Thirumdas et al. 2018; Gott et al. 2023).

There are various methods for producing PAW using different plasma generation technologies, with the most commonly used approach involving a plasma jet, where electric discharges are directly applied to the water (Thirumdas et al. 2018; Oliveira et al. 2022; Hadinoto et al. 2023). In this study, we utilised a plasma bubble reactor (PBR) to generate PAW from sterilised tap water. As mentioned in a study by Hadinoto et al. (2023) this method of PAW production was found to be more effective, as the interaction of plasma bubbles with water minimizes the loss of reactive species and enhances their dissolution into the liquid. To our knowledge, research on the antimicrobial activity of PAW produced using plasma bubbles remains limited, as most studies have focused on alternative PAW production methods. The aim of our study was to evaluate the physicochemical properties of PAW and their stability during storage, such as pH, electrical conductivity, and concentrations of key RONS, and its antimicrobial efficacy against common foodborne pathogens, *E. coli* O157 and *S. aureus*. PAW’s inactivation efficiency was assessed against both planktonic and biofilm states. Furthermore, considering reports that disinfectant activity is often inhibited by organic contamination (Yemmireddy and Hung 2015; Teng et al. 2018; Yokoyama et al. 2021; Şahiner et al. 2022), we examined the effect of organic matter on the decontamination activity of PAW. In addition, we examined the antimicrobial efficacy of PAW after 24 h and 48 h of storage, including under organic load conditions, to better understand its stability and applicability over time. Lastly, given the potential contact of PAW with human skin during application, we investigated its cytotoxic effects on human keratinocytes to assess its safety as a novel disinfectant.

To our knowledge, this is the first study to comprehensively evaluate PAW generated using a potentially scalable PBR under conditions relevant to real-world agri-food environments. Unlike many previous investigations relying on plasma jets and sterile distilled water (Xiang et al. 2019; Wang et al. 2023; Shen et al. 2016), our study employed autoclave-sterilised tap water and atmospheric air to generate PAW, offering a more practical and scalable solution. We assessed PAW’s antimicrobial efficacy against both Gram-negative and Gram-positive pathogens in planktonic and biofilm states, systematically investigated the interference of organic matter, and evaluated cytotoxicity on human keratinocytes within a single, integrated framework. Importantly, we also examined the antimicrobial properties of stored PAW (after 24 and 48 h at 4 °C), including its effectiveness against bacterial biofilms in the presence and absence of organic matter, an aspect that, to our knowledge, has not been previously investigated for PAW produced via a PBR.

## Materials and methods

### Bacterial cultivation

The antibacterial efficacy of PAW was tested against *Staphylococcus aureus* (NCTC 12981) and a non-Shiga toxin-producing *Escherichia coli* serotype O157, designated (NCTC 12900). Bacterial cultures were initially grown on Tryptone Soya Agar with 0.6% yeast extract (TSAYE, Oxoid, UK) and incubated at 37 °C for 24 h. Subsequently, the cultures were preserved on slants of the same media at 4 °C for further use.

### Plasma-activated water generation and treatment

**Fig. 1:**
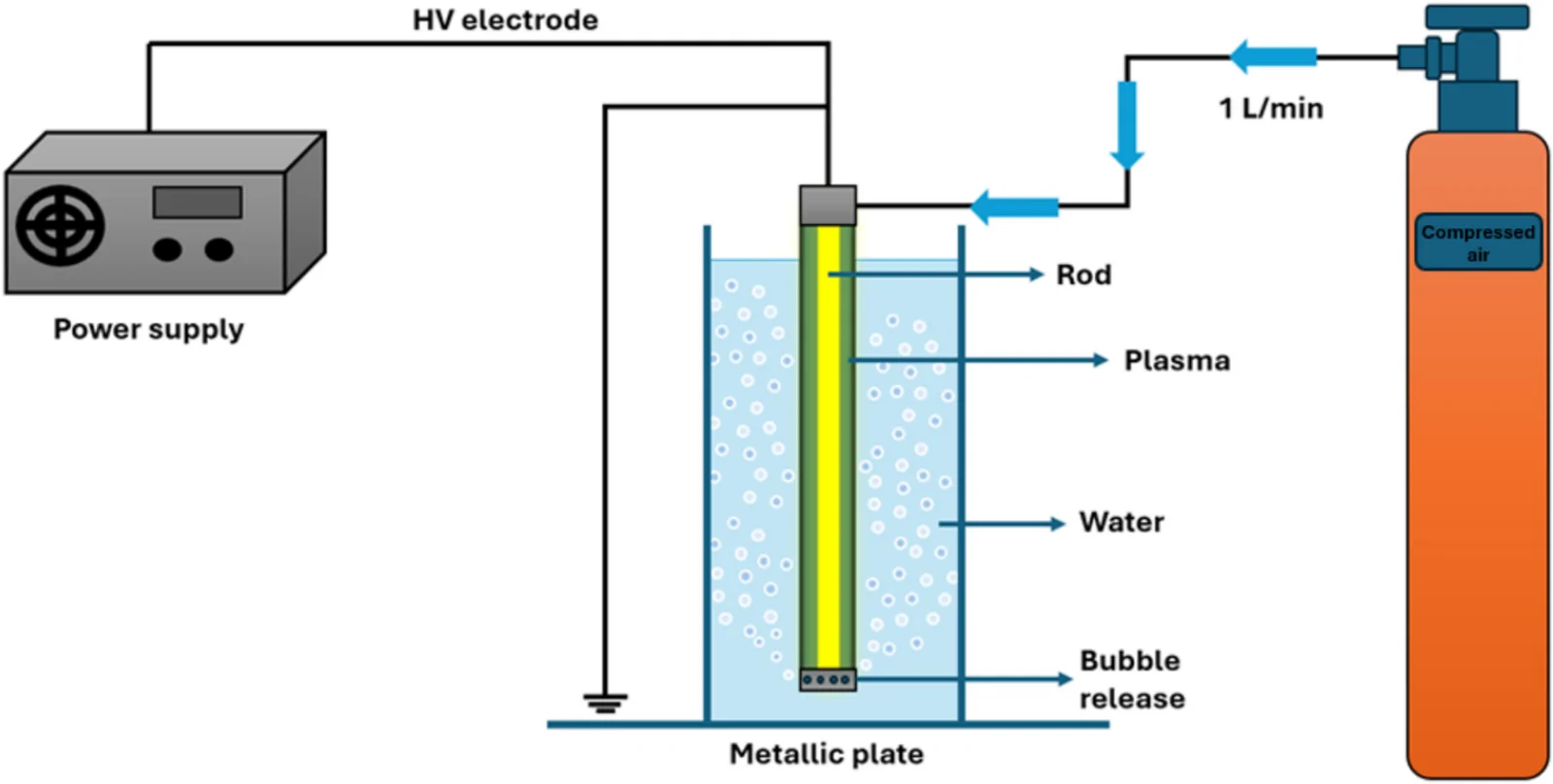
Schematic representation of the plasma-activated water generation experimental set-up. This includes a cylinder with compressed air, a plasma bubble reactor, consisting of an acrylic tube submerged in water and a high‐voltage power supply. Blue arrows indicate the flow of the feed gas at a flow rate of 1L/min

A plasma bubble reactor was used to generate PAW. Figure 1 depicts the configuration of PBR, where the PBR was submerged in 100 mL of sterile tap water inside Schott bottles. The reactor comprised an acrylic cylinder measuring 140 mm in length, sealed at both ends with machined caps. Each cap supports a 4-mm stainless steel rod, aligned along the cylinder's axis, serving as high-voltage electrodes. On the outer surface of the grounded electrode, a 5-mm-wide strip of adhesive copper tape connects to the plasma power supply's ground wire.

Plasma formation occurred under ambient air conditions, powered by a high voltage supply (PlasmaLeap Technologies). PAW was generated for 20 min at 150 V, and two discharge frequencies were used: 1000 Hz and 1500 Hz. The acrylic cylinder was immersed in water and featured ten 2-mm holes drilled 8 mm above the base, allowing the discharge of electrically charged bubbles into the water. Compressed air was introduced into the PBR at a rate of 1 L/min through a tube at the opposite end of the cylinder. As the bubbles escape through these holes, the reactive species within them interact with the water at the bubble-water interface where the mass transfer takes place. For the control experiment, 100 mL of autoclave-sterilised tap water was subjected to air flow at 1 L/min without plasma discharge.

To ensure consistency and minimize variability across all experiments, the same batch of autoclave-sterilized tap water was used for PAW production throughout the study.

### Physicochemical characterization of PAW after production and during storage

Following the production and storage of PAW, parameters pH, electrical conductivity (EC), hydrogen peroxide (H_2_O_2_) concentration, nitrite (NO₂⁻) concentration, and nitrate (NO₃⁻) concentration were evaluated. EC and pH levels were determined using a conductivity meter (Jenway 4200) and a pH meter (FiveEasy, Mettler Toledo), respectively. Concentration measurements of H_2_O_2_, NO₂⁻, and NO₃⁻ followed methodologies referenced in Asimakopoulou (2022). The measurement of H_2_O_2_ employed the titanium oxysulfate (TiOSO_4_; Sigma-Aldrich) technique, with subsequent spectrophotometric analysis for quantification.

For nitrite levels, the Griess reaction was utilized, involving N-(1-naphthyl)ethylenediamine hydrochloride and sulfanilic acid to produce a magenta azo dye. Nitrate levels were assessed using a commercial nitrate assay kit (Sigma), which relies on the compound's reaction with 2,6-dimethylphenol (DMP). To eliminate nitrite interference in the nitrate measurements, all PAW samples underwent a pretreatment with sulfamic acid before testing.

### PAW treatment and enumeration of S. aureus and E. coli 0157 grown in planktonic mode

Bacterial suspensions of planktonic cells were prepared from overnight cultures, which were then centrifuged at 6500 rpm for 10 min. The cell pellets were washed and resuspended in sterile phosphate-buffered saline (PBS), followed by two additional rounds of centrifugation and washing, to remove any residual organic load. Planktonic *S. aureus* and *E. coli* O157 cultures were prepared by inoculating 96-well microtiter plates with 150 μL each bacterial strain, achieving an initial concentration of 5 log CFU/mL. After 1 h, 200 μL PAW, produced for 20 min at either 1000 Hz or 1500 Hz or control water (without plasma discharge),, was added to the wells. The bacterial suspensions were then exposed to PAW for 5, 10, 15 and 20 min (treatment). To enumerate surviving bacterial cells, suitable tenfold dilutions were prepared. An aliquot of 100‐μl was used from the appropriate tenfold serial dilutions and was spread plated on Trypticase Soy Agar (TSA, Oxoid). Plates were incubated at 37 °C for 24 h, and planktonic bacterial cells were expressed as log CFU/ml. To assess the impact of organic matter, bovine serum albumin (BSA) was added to wells containing bacteria at concentrations of 0–0.1 g/L to simulate organic contamination, prior to treatment with PAW for 20 min. The experiment was repeated under these conditions.

### S. aureus and E. coli biofilm preparation and enumeration

*S. aureus* and *E. coli* biofilms were formed separately at the bottom of 96-well microtiter plates. Each well was inoculated with 150 μL of bacterial culture (6 log CFU/ml) and incubated at 37 °C for 48 h to allow for the formation of biofilms. Prior to PAW or control treatment, biofilms were washed with sterile PBS to remove loosely attached planktonic cells and surface debris. Following PAW treatment, biofilms were washed once with 200 μL of PAW or control water for different exposure times (5, 10, 15, or 20 min). PAW (or control) was then removed from the wells, and the biofilms were washed, resuspended in sterile PBS and subjected to tenfold serial dilutions, followed by spread plating on TSA and incubation overnight at 37 °C for subsequent CFU/mL determination. To assess the effect of organic load on PAW antimicrobial activity against biofilm cells, different concentrations of 0—0.1 g/L of bovine serum albumin (BSA) were added to the initial cultures to simulate the presence of organic material, and the experiments were repeated as above in six replicates.

The antimicrobial activity of stored PAW was assessed using the same procedure described above. When testing stored PAW in the presence of organic matter, only one concentration of BSA (0.01 g/L) was used, as this was sufficient to demonstrate the effect of organic load on the antibacterial activity of PAW.

### Human keratinocytes (HaCaT) cytotoxicity assay

Human keratinocytes (HaCaT) cells were cultured in Dulbecco’s Modified Eagle Medium (DMEM) (Merck, UK), supplemented with 2 mM L-glutamine and 10% fetal bovine serum at 37 °C. To develop HaCaT cell monolayers, the methodology described by Vyas et al. (2023) was employed, enhancing the skin epithelial cell model with host factors. The process involved coating 96-well microtiter plates with 300 μg/mL of collagen I (Merck) for one hour at 37 °C. Following the collagen treatment, the plates were cleared of excess collagen and washed with sterile (PBS). Each well was subsequently seeded with a suspension of HaCaT cells at a concentration of 1 × 10⁶ cells/mL and incubated at 37 °C.

The MTT assay (3-(4,5-dimethylthiazol-2-yl)−2,5-diphenyltetrazolium bromide) was used to evaluate cell viability post-exposure to PAW. Treatment conditions included: control (untreated), PAW exposure for 15, 30, and 45 min, hydrogen peroxide (positive control for cytotoxicity). The assay followed Merck’s protocol, as outlined by Marches et al. (2022).

### Statistical analysis

Statistical analysis was conducted using GraphPad Prism (version 10.2.2 (397) for Windows GraphPad Software, Boston, Massachusetts, USA, www.graphpad.com). One-way analysis of variance (ANOVA), with subsequent Tukey’s multiple comparisons test, was employed to determine group-wise differences. A two-way ANOVA was also employed to determine differences in the susceptibility of *E. coli* and *S. aureus* to PAW produced at different discharge frequencies. Statistical significance was set at p < 0.05. Each treatment was done six times (two sets, with three replicates within each set), except for HaCaT viability assays, which were done in three replicates.

## Results

### Physicochemical properties of PAW

To determine the stability of PAW, its physicochemical properties, pH, EC, and RONS concentrations, were measured immediately after production and after 24 and 48 h of storage at 4 °C (Table 1).

**Table 1:** Physicochemical properties of PAW generated from autoclave sterilised tap water and stored for different periods. Data are presented as mean ± SD. Each measurement was performed in triplicate, with each set replicated twice, resulting in n = 6. Different letters within the same column indicate significant differences (p < 0.05)

Storage time	pH	Conductivity (μS/cm)	H_2_O_2_ mg/L	NO_2_^−^ mg/L	NO_3_^−^ mg/L
Control	7.37 ± 0.03^a^	235 ± 3.61^a^	0.0 ± 0.0^a^	0.0 ± 0.0^a^	0.0 ± 0.0^a^
0h*	6.1 ± 0.05^b^	309.17 ± 9.37^b^	26.51 ± 2.64^b^	35.78 ± 1.65^b^	37.21 ± 2.17^b^
24h	6.08 ± 0.05^b^	308 ± 8.44^b^	24.16 ± 2.37^b,c^	31.73 ± 1.06^c^	33.66 ± 1.46^c^
48h	6.06 ± 0.04^b^	308.67 ± 4.84^b^	22.86 ± 2.48^c^	28.15 ± 1.84^d^	30.04 ± 1.23^d^

* *PAW characteristics straight after production*

The pH significantly decreased after activation, while conductivity increased; however, both parameters remained stable without significant changes during 48 h of storage. Measurements for these indicators don’t significantly differ from freshly produced PAW. Freshly produced PAW contained significantly higher levels of hydrogen peroxide compared to untreated water; a slight but statistically significant decline in H₂O₂ concentration was observed after 24 h and 48 h of storage. Additionally, NO₂⁻ and NO₃⁻ concentrations increased substantially after plasma activation. However, nitrite and nitrate levels also dropped significantly after 24 and 48 h of storage. The NO₂⁻ level decreased from 35.78 mg/L to approximately 28.15 mg/L, while NO₃⁻ dropped from 37.21 mg/L to 30.04 mg/L.

### Antibacterial activity of PAW against planktonic E. coli and S. aureus

**Fig. 2:**
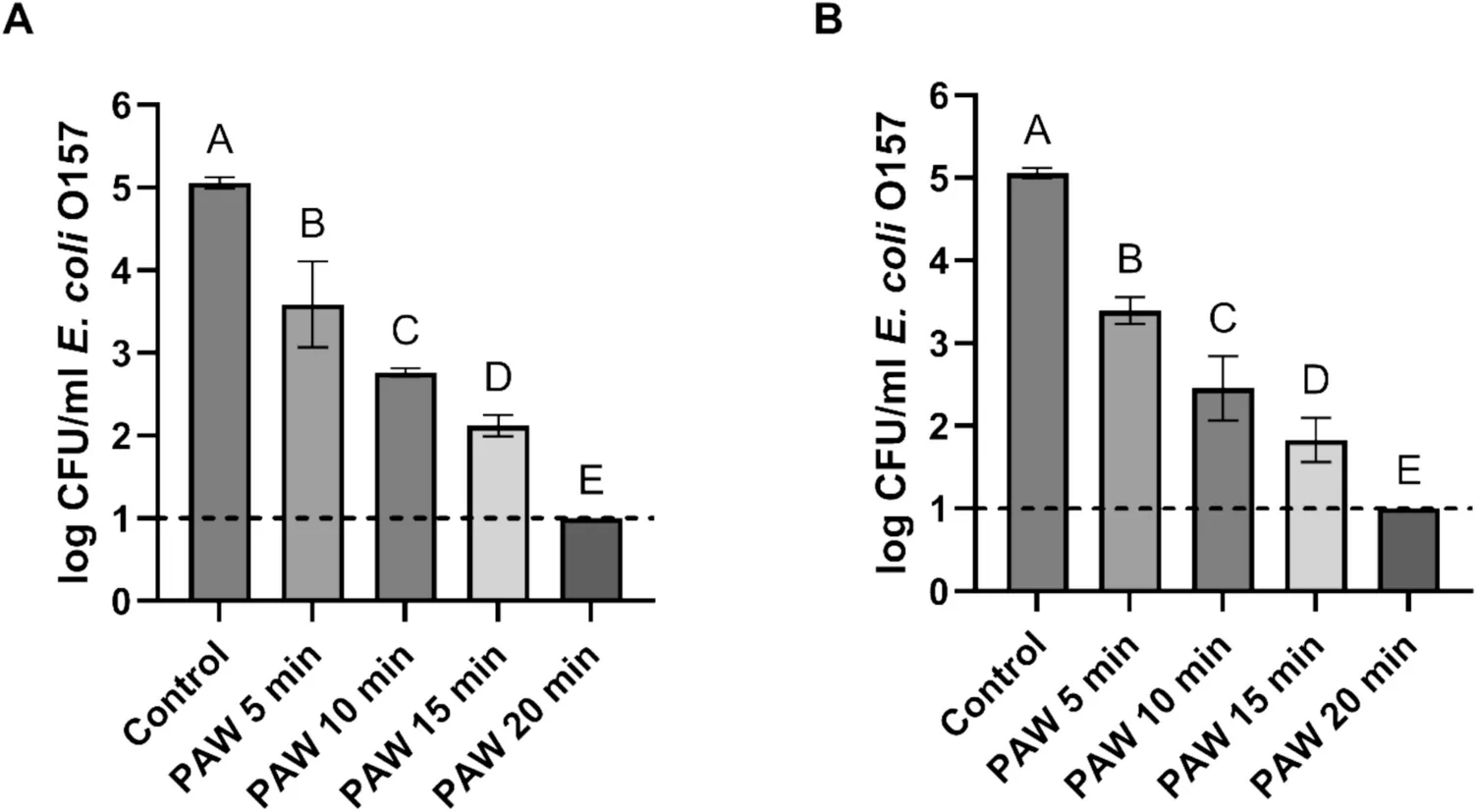
Viable counts of *E. coli* planktonic cells after treatment with PAW produced at 150 V & 1000 Hz (Graph A) and 150 V & 1500 Hz (Graph B) for different treatment durations: 5, 10, 15, and 20 min. Each bar represents the mean value, with standard deviation indicated by error bars. Each treatment was performed in triplicate, with each set replicated twice, resulting in n = 6. Different letters above the bars indicate significant differences between treatments (p < 0.05). The dotted line indicates the detection limit (1 log CFU/ml)

**Fig. 3:**
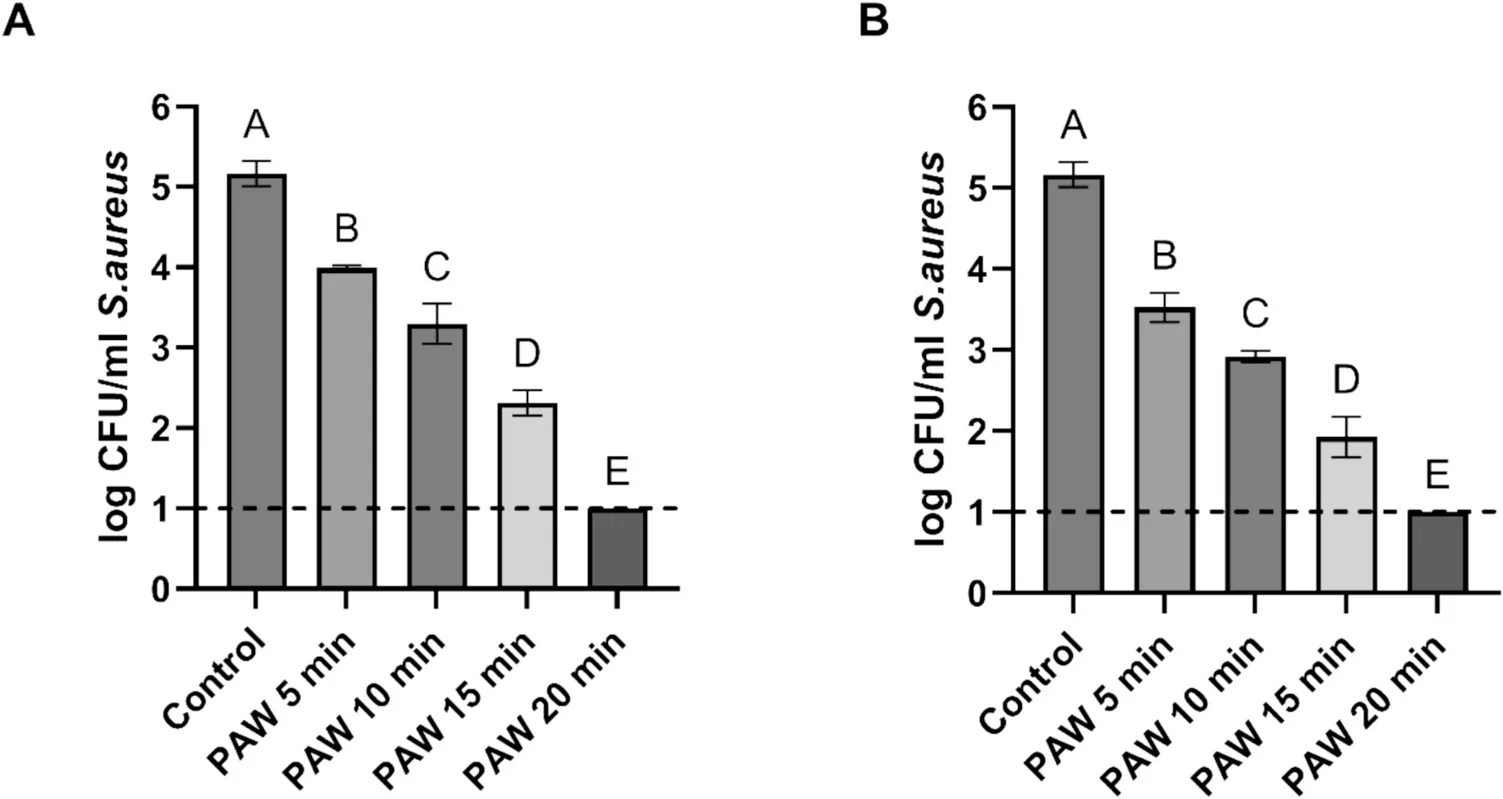
Viable counts of *S. aureus* planktonic cells after treatment with PAW produced at 150 V & 1000 Hz (Graph A) and 150 V & 1500 Hz (Graph B) for different treatment durations: 5, 10, 15, and 20 min. Each bar represents the mean value, with the standard deviation (SD) indicated by error bars. Each treatment was performed in triplicate, with each set replicated twice, resulting in n = 6. Different letters above the bars indicate significant differences between treatments (p < 0.05).). The dotted line indicates the detection limit (1 log CFU/ml)

First, we aimed to identify the most effective settings for PAW production. PAW was generated at 150 V & 1000 Hz and 150 V & 1500 Hz and tested against two common foodborne bacterial pathogens: *E. coli* and *S. aureus*. Planktonic cells of both pathogens were treated with PAW for 5, 10, 15 and 20 min, followed by plating to assess the antibacterial effects of PAW produced at different discharge frequencies. The results for *E. coli* and *S. aureus* treatment are shown in Figs. 2 and 3, respectively.

As shown in Fig. 2, the initial *E. coli* population in untreated water (control) was approximately 5 Log CFU/mL. After 5 min of PAW treatment, the viable bacterial counts decreased to 3.59 ± 0.52 Log CFU/mL and 3.39 ± 0.16 Log CFU/mL at 1000 Hz and 1500 Hz discharge frequencies, respectively. Prolonged treatment durations of 10 and 15 min resulted in further significant reductions. Extending the exposure time to 20 min led to a reduction of bacterial counts below the detection limit for both discharge frequencies.

The results for *S. aureus*, presented in Fig. 3, followed a similar pattern to those observed for *E. coli*. Even the shortest exposure time of 5 min reduced viable counts from approximately 5 Log CFU/mL to 3.99 ± 0.03 Log CFU/mL at 1000 Hz and 3.53 ± 0.18 Log CFU/mL at 1500 Hz. Increasing treatment durations to 10 and 15 min further reduced viable counts. After 20 min of exposure to PAW, the viable counts of *S. aureus* fell below the detection limit of 1 log CFU/mL for both discharge frequencies.

### Effect of organic load on PAW antimicrobial activity against S. aureus and E. coli in the planktonic state

Organic contamination can often affect the efficacy of disinfectants (Şahiner et al. 2022). In this study, we examined how different concentrations of added organic load, in the form of bovine serum albumin (BSA), affect the efficiency of PAW against pathogens. Based on the results described above, a discharge frequency of 1500 Hz for PAW production and an exposure time of 20 min was used for subsequent experiments, as it showed the highest antimicrobial effect.

**Fig. 4:**
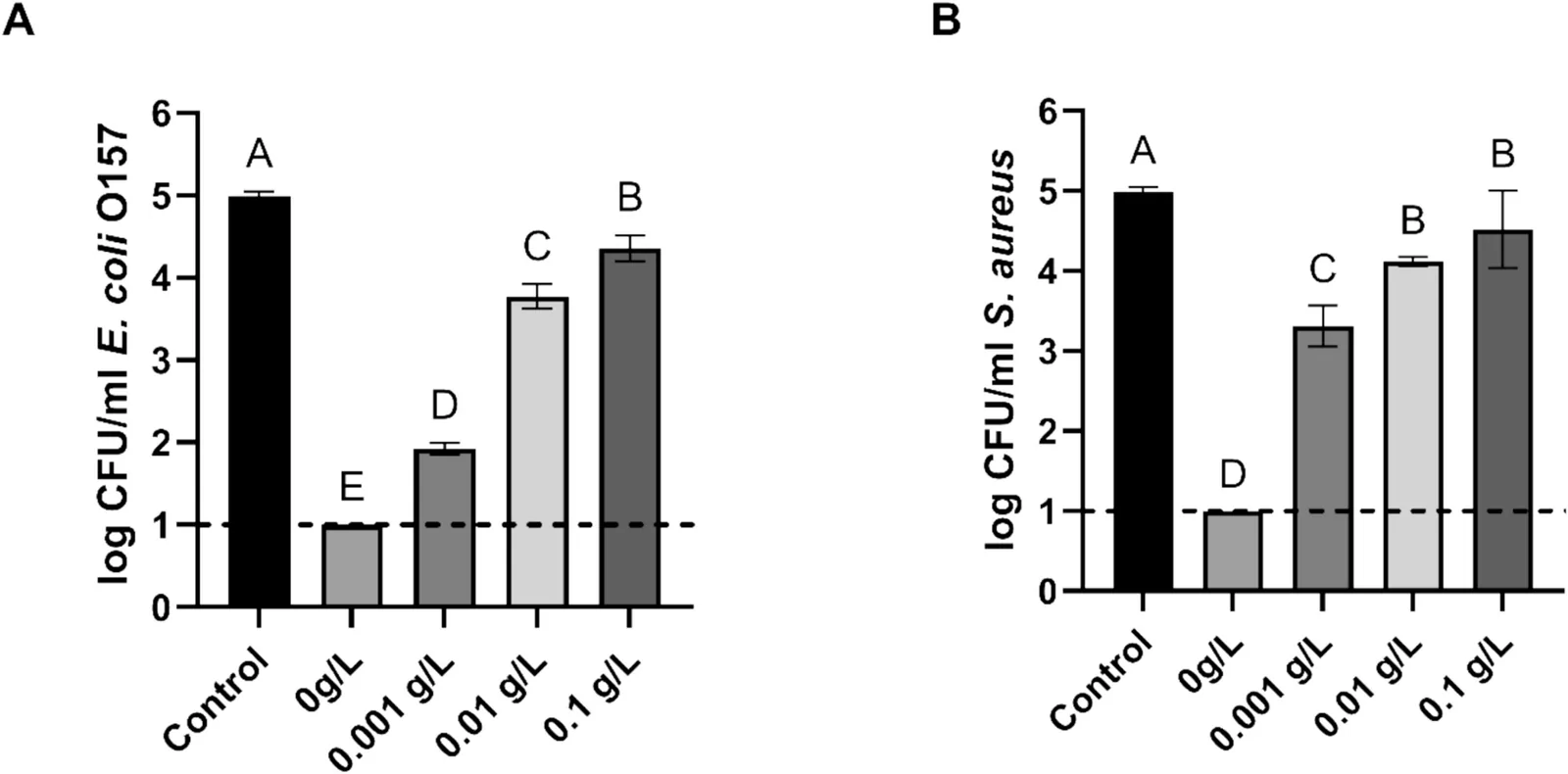
Viable counts of bacterial planktonic cells after treatment with PAW for 20 min in the presence of organic load: *E. coli* (Graph A), *S. aureus* (Graph B). Each bar represents the mean value, with the standard deviation indicated by error bars. Each treatment was performed in triplicate, with each set replicated twice, resulting in n = 6. Different letters above the bars indicate significant differences between treatments (p < 0.05). The dotted line indicates the detection limit (1 log CFU/ml)

Initial cultures of *E. coli* and *S. aureus* were supplemented with BSA at concentrations of 0, 0.001, 0.01, and 0.1 g/L before being treated with PAW for 20 min. The results are shown in Fig. 4. Consistent with the previous experiment, treatment of planktonic bacterial cells with PAW for 20 min, without added BSA, led to complete bacterial inactivation. However, even the lowest concentration of BSA (0.001 g/L) reduced the antibacterial efficacy of PAW, with bacterial counts exceeding the detection limit. *E. coli* (1.92 ± 0.07 log CFU/mL) and *S. aureus* (3.31 ± 0.26 log CFU/mL) showed significantly reduced PAW sensitivity.. Notably, *S. aureus* exhibited higher viable counts than *E. coli* at this concentration, suggesting a greater reduction in PAW efficacy against the Gram-positive strain.

Higher BSA concentrations further diminished PAW’s antibacterial activity. PAW treatment in the presence of 0.01 g/L and 0.1 g/L BSA resulted in log reductions of 1.22 and 0.63 for *E. coli*, and 0.87 and 0.47 for *S. aureus*, respectively, compared to the initial cell concentrations in the control group. Significant differences (p < 0.05) were observed between all treatment and control groups, except for the *S. aureus* samples treated with 0.01 g/L and 0.1 g/L BSA, which showed no statistically significant difference.

## Antimicrobial activity of PAW against ***E. coli*** and ***S. aureus*** in biofilm state.

**Fig. 5:**
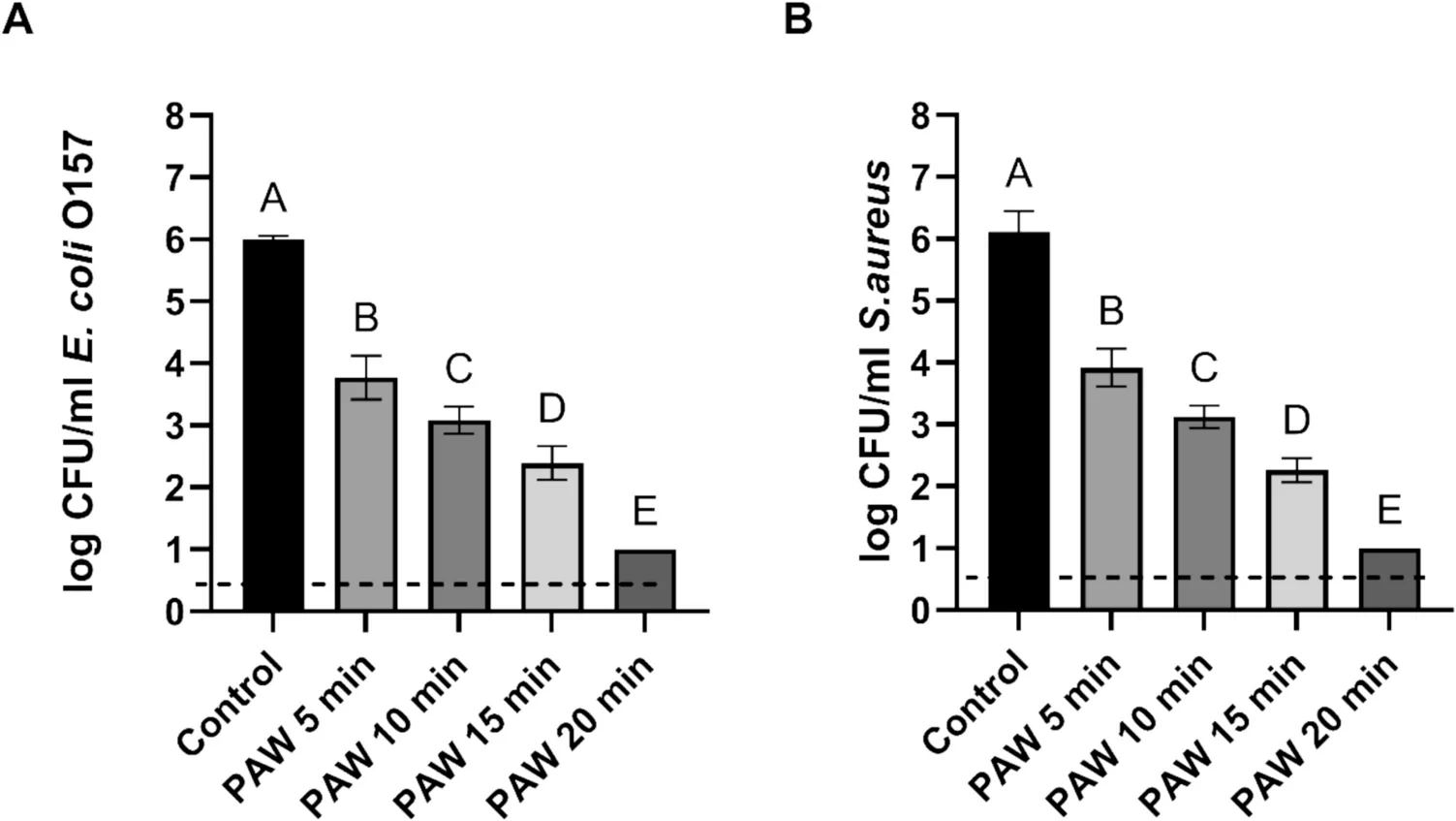
Biofilm survival of *E. coli* (Graph A) and *S. aureus* (Graph B) following PAW treatment. Each bar represents the mean value, with the standard deviation indicated by error bars. Each treatment was performed in triplicate, with each set replicated twice, resulting in n = 6. Different letters above the bars indicate significant differences between treatments (p < 0.05). The dotted line indicates the detection limit (1 log CFU/ml)

Biofilm viability reduction was evaluated over 5–20 min of PAW exposure (Fig. 5). For *E. coli* biofilms a significant antimicrobial effect was observed at all treatment points: 5, 10, 15, and 20 min, it was reduced to 3.77 (2.23 log reduction), 3.08 (2.92), 2.39 (3.61), and below 1 log (> 5 log reduction), respectively, compared with cells counts in untreated sample 6 log CFU/ml. Similarly, *S. aureus* biofilms showed a progressive reduction in viable cell counts following PAW treatment. Cell counts dropped to 3.92 (2.19 log reduction) after 5 min, 3.12 (2.99) after 10 min, 2.26 (3.85) after 15 min and consequently dropped under the detection limit (> 5 log reduction) after 20 min of exposure to PAW. The control group maintained a viable cell count at 6.11 log CFU/ml. A significant difference (p < 0.05) was found between all treatment and control groups for both *E. coli* and *S. aureus* biofilms.

### Effect of organic load on PAW antimicrobial activity against S. aureus and E. coli in the biofilm state

**Fig. 6:**
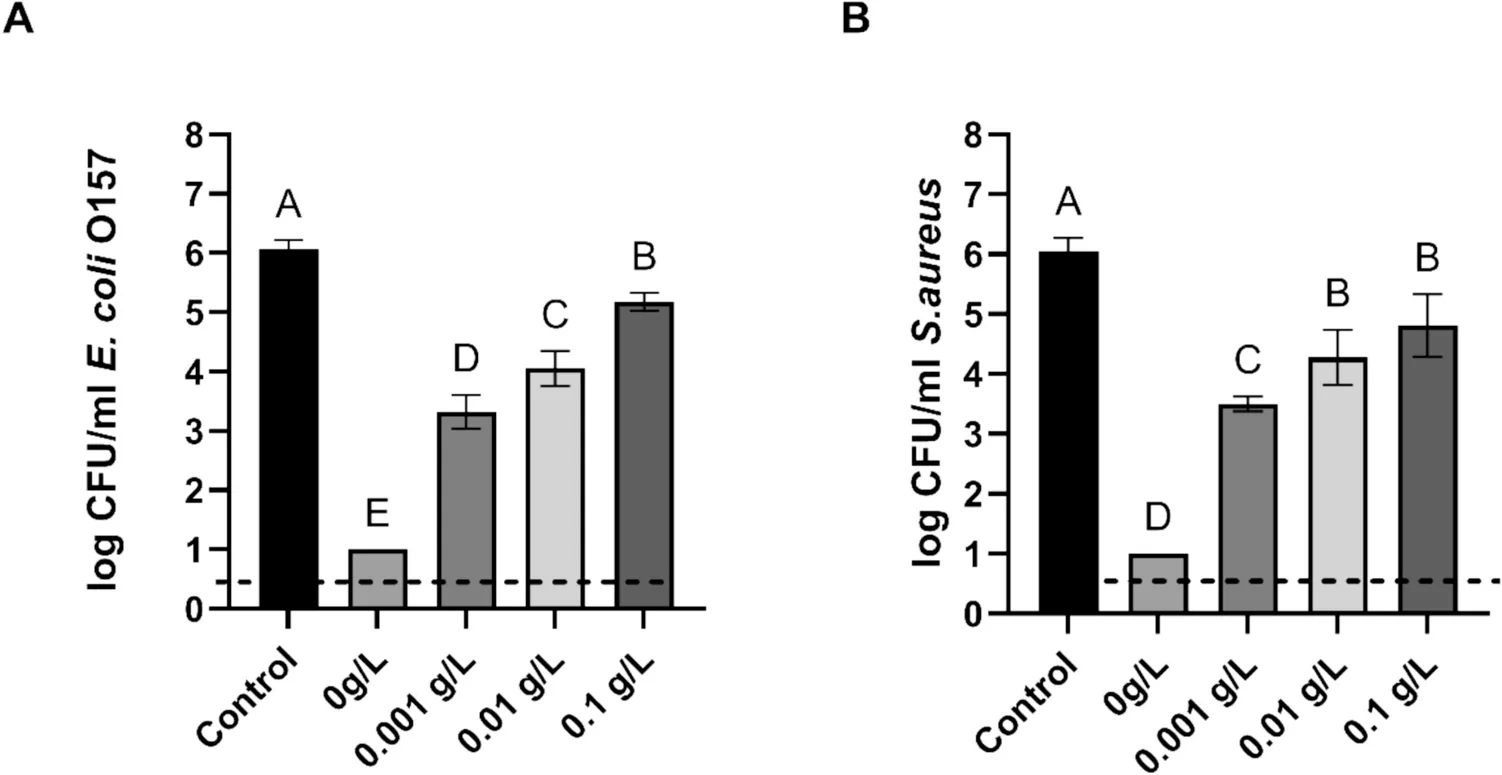
Biofilm survival of *E. coli* (Graph A) and *S. aureus* (Graph B) after PAW treatment in the presence of organic load. Each bar represents the mean value, with the standard deviation indicated by error bars. Each treatment was performed in triplicate, with each set replicated twice, resulting in n = 6. Different letters above the bars indicate significant differences between treatments (p < 0.05). The dotted line indicates the detection limit (1 log CFU/ml)

PAW’s biofilm inactivation was negatively impacted by organic load (Fig. 6). Exposure to PAW alone for 20 min reduced the initial populations of *E. coli* and *S. aureus* below the detection limit. However, the presence of BSA at concentrations of 0.001, 0.01, and 0.1 g/L significantly reduced the inactivation levels, resulting in a decrease for *E. coli* biofilms by 2. 74, 2.05 and 0.92 log CFU/ml, respectively. Reduction in effectiveness followed the same trend for *S. aureus* biofilms. The following treatment with PAW and added BSA at 0.001, 0.01, and 0.1 g/L led to a decrease in *S. aureus* viable counts by 2.54, 1.76 and 1.24 log CFU/ml, respectively. Significant differences (p < 0.05) were observed between all treatment and control groups, except for the *S. aureus* biofilms treated with 0.01 g/L and 0.1 g/L BSA, which showed no statistically significant difference.

### Antimicrobial activity of stored PAW against S. aureus and E. coli biofilms in the absence and presence of organic matter

To further evaluate PAW’s stability after storage at 4 °C for 24 and 48 h, we additionally assessed its antimicrobial activity against *S. aureus* and *E. coli* biofilms, in addition to its physicochemical properties discussed previously.

Extended storage led to a significant reduction in antimicrobial effectiveness against both pathogens (Table 2). Notably, PAW stored for 24 h remained as effective as freshly produced PAW in inactivating *E. coli* biofilms. However, its efficacy against *S. aureus* declined, as evidenced by a significant increase in viable cell counts from 2.12 log CFU/ml (fresh PAW) to 2.7 log CFU/ml following treatment with PAW stored for 24 h.

**Table 2:** Antimicrobial activity of PAW generated from autoclave-sterilised tap water and stored for different durations, tested in the presence and absence of organic matter under a 15-min exposure. Data are presented as mean ± SD in CFU/ml. Each measurement was performed in triplicate, with each set replicated twice, resulting in n = 6. Different letters indicate significant differences (p < 0.05)

Bacteria		PAW storage time		
	NT^*^	0h	24h	48h
*E. coli*	5.83 ± 0.22^a^	2.72 ± 0.15^b^	2.53 ± 0.2^b,d^	3.73 ± 0.24^c^
*S. aureus*	5.91 ± 0.19^a^	2.12 ± 0.13^b^	2.7 ± 2.28^d^	3.52 ± 0.26^c^
*E. coli* + BSA^**^	6.07 ± 3.84^a^	3.84 ± 0.16^c^	4.77 ± 0.18^e^	5.24 ± 0.15^f^
*S. aureus* + BSA^**^	6.12 ± 0.11^a^	3.79 ± 0.17^c^	4.44 ± 0.33^e^	5.33 ± 0.14^f^

*NT: non-treated (control) **BSA was added at single concentration (0.01 g/L) to simulate organic contamination

The presence of BSA at a concentration of 0.01 g/L further reduced the antimicrobial activity of stored PAW, resulting in comparable levels of bacterial inactivation for both pathogens.

### Effect of PAW on human keratinocyte (HaCaT) viability

**Fig. 7:**
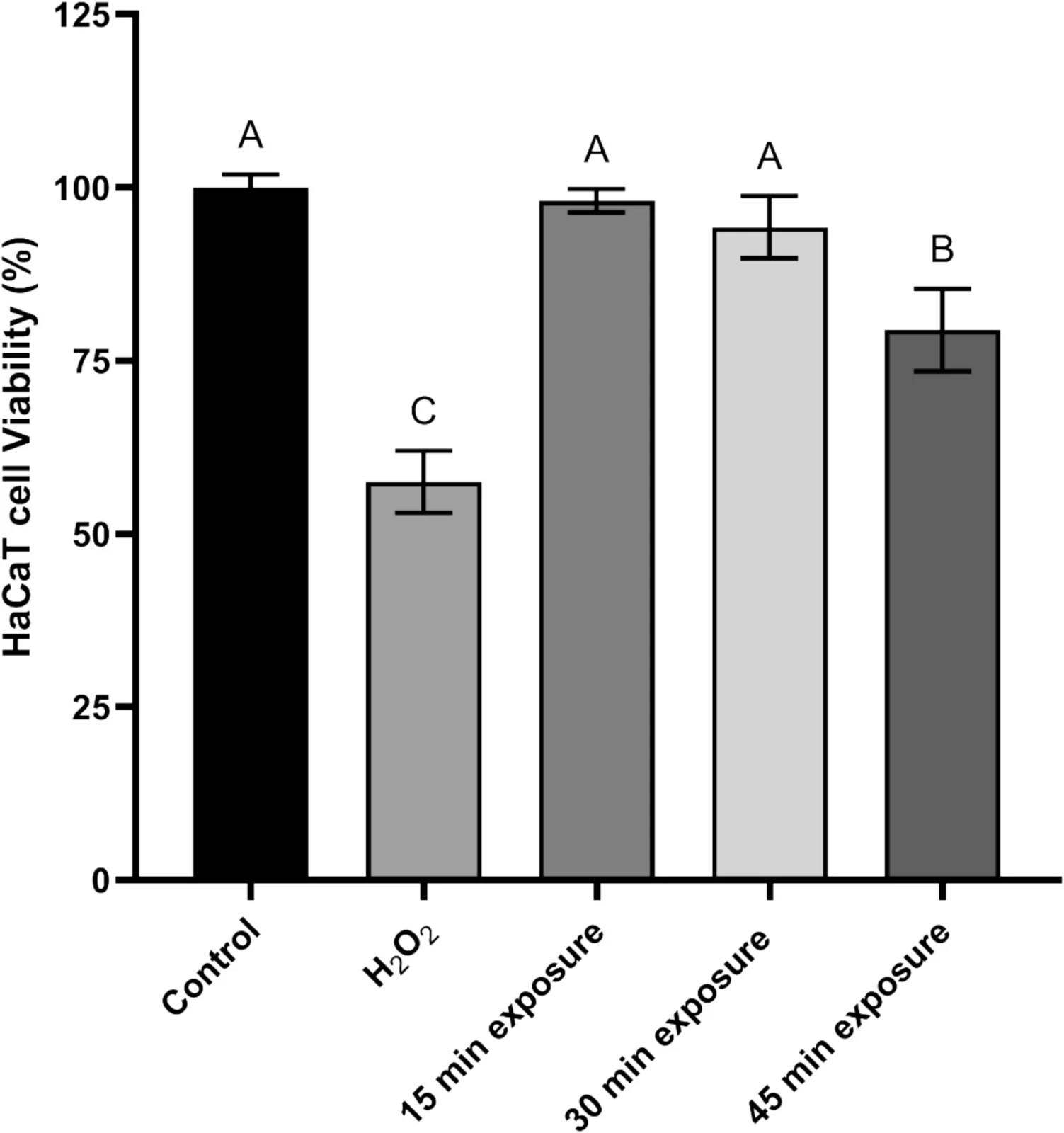
HaCaT cell viability after PAW and hydrogen peroxide exposure. Each bar represents the mean value, with standard deviation indicated by error bars, calculated from three replicates. Different letters above the bars indicate significant differences between treatments (p < 0.05)

PAW cytotoxicity was assessed on HaCaT cells at 15, 30, and 45 min of exposure (Fig. 7). Two controls were used: untreated normal skin cells as the negative control and a positive control where hydrogen peroxide was used as a cytotoxic agent, resulting in approximately 43% of the cell death.

Quantification of human keratinocytes after PAW treatment revealed no significant difference between treated and untreated groups for exposure durations of 15 and 30 min, with cell death rates of approximately 2 and 6 per cent, respectively. However, treatment for 45 min led to a significant reduction in cell viability (p < 0.05) compared to the initial cell count and shorter treatment durations, with 21% of cells dying.

## Discussion

PAW has potential as a sustainable disinfectant with potential applications in food safety, healthcare, and environmental sanitation (Rahman et al. 2022; Oliveira et al. 2022; Han et al. 2023). It offers key advantages over traditional chemical disinfectants, including low energy consumption, cost-effectiveness, and an environmentally friendly production process that eliminates the need for synthetic chemicals (Oliveira et al. 2022). In this study, atmospheric air and tap water was used to produce PAW, both of which are readily available and inexpensive resources. The use of atmospheric air, rather than expensive or high-purity gas mixtures, not only simplifies the process but also contributes to the sustainability and scalability of the technology. Similarly, the use of tap water as opposed to ultra-pure or laboratory-grade water demonstrates the practicality of PAW generation under real-world conditions, supporting its feasibility for large-scale applications without the need for specialized infrastructure or purification systems.

Recent studies have demonstrated PAW’s antimicrobial activity against a range of bacterial foodborne pathogens, highlighting its effectiveness, and application versatility (Zhao et al. 2020; Rothwell et al. 2022; Moonsub et al. 2024; Měřínská et al. 2025). Here, we tested the antimicrobial properties of PAW produced from tap water using a PBR under two different generation conditions. Additionally, we evaluated the physicochemical characteristics of PAW produced at 1500 Hz discharge frequency and how these properties changed after storage. The storage of PAW for 24 and 48 h did not affect its pH and electrical conductivity, when stored at 4 °C. However, we also measured the levels of key reactive species responsible for microbial deactivation, namely, hydrogen peroxide, nitrate, and nitrite (Zhou et al. 2020). Hydrogen peroxide levels showed a slight yet statistically significant decline after 24 and 48 h of storage. In contrast, nitrate and nitrite levels decreased more substantially over the same storage period, with notable reductions observed already after 24 h and continuing to decline by 48 h. Wang et al. (2023) reported that the pH of PAW remained stable for 72 h when stored at 4 °C and 22 °C. Nevertheless, they also observed a fluctuation in relative conductivity and a dramatic decrease in nitrate and nitrite concentrations, which had a significant impact on the inactivation activity of PAW (Wang et al. 2023). In a study by Shen et al. (2016), PAW stored for 30 days at 25 °C, 4 °C, −4 °C, and −80 °C was found to have a stable pH with slight variations, while NO₃⁻ levels gradually decreased over time regardless of the storage temperature. In contrast, NO₂⁻ and H₂O₂ levels decreased as storage temperature increased but remained relatively stable at −80 °C, preserving PAW's antimicrobial activity against *S. aureus*. Unlike our study, which used tap water, the studies mentioned above used sterile distilled water to produce PAW. However, regardless of the type of water used, the levels of the reactive species decreased after storage, affecting the antibacterial properties of PAW.

In this study, freshly produced PAW, generated at discharge frequencies 1000 Hz and 1500 Hz, was tested against *E. coli* and *S. aureus*. Our results demonstrated that inactivation levels for planktonic cells of both pathogens increased progressively with the PAW exposure time, resulting in inactivation below the detection limit (1 log CFU/ml) of bacteria after 20 min of treatment. The inactivation patterns indicated that a higher discharge frequency of 1500 Hz was more effective than 1000 Hz. At 1000 Hz, bacterial counts decreased by 2.94 Log CFU/ml for *E. coli* and 2.86 Log CFU/ml for *S. aureus* after exposure to water treated with CAP at 1000 Hz. When the discharge frequency was increased to 1500 Hz, the reductions were significantly higher, with viable cell counts declining by > 3 Log CFU/mL for both pathogens. Furthermore, higher discharge frequency was found to be more effective in producing PAW with strong antibacterial activity, as demonstrated by Hadinoto et al. (2021). They showed that PAW generated using a pinreactor with a discharge frequency of 1000 Hz was significantly less effective against *E. coli* and *S.* Typhimurium planktonic cells than water activated at a higher frequency. Similar findings were reported by Rothwell et al. (2022), who tested three discharge frequencies (500, 1000, and 1500 Hz) for PAW production. The highest discharge frequency (1500 Hz) was the most effective, enhancing the antimicrobial activity of PAW against *E. coli* and *Listeria*.

Bacterial pathogens in the form of biofilms are known to be more resistant to disinfectants and other disinfection treatments (Bridier et al. 2011). This increased resistance is attributed to their unique structure, which includes extracellular polymeric substances (EPS). These compounds, produced by bacteria, form an additional protective layer that shields the bacteria from external stimuli and encases them in a clumped arrangement (Mai-Prochnow et al. 2021). Biofilm formation presents a significant challenge in various settings. As a result, considerable attention has been devoted to testing novel disinfectants on biofilm-forming pathogens (Zhao et al. 2023a; Fernandes et al. 2024). Therefore, the effectiveness of PAW was also tested on biofilms. Overall, *E. coli* and *S. aureus* biofilms exhibited a similar inactivation pattern after treatment with PAW generated at 1500 Hz for 20 min as planktonic cells. Although the initial cell counts in biofilms were approximately 1 log higher than those in planktonic populations, both pathogen biofilm levels were reduced below the detection limit following the longest PAW treatment (20 min). Shorter treatment durations, with a 5-min difference, also resulted in significant decreases in viable cell counts across all treatment groups. These findings are consistent with the results of the previous experiment in our study, further confirming the high antibacterial activity of the PAW produced. Vyas et al. (2023) studied PAW's activity against *E. coli* biofilms and its mechanism of anti-biofilm action. To produce PAW, they used a bubble spark discharge reactor and autoclave sterilised Milli-Q water. Their findings revealed that PAW's temperature and pH did not significantly affect biofilm viability, suggesting that reactive oxygen and nitrogen species play a primary role in biofilm destruction. Another study by Tan and Karwe (2021) revealed that PAW was as effective as chlorine water at a free chlorine concentration of 100 ppm, reducing the *Enterobacter aerogenes* population by approximately 3.5 Log CFU/cm^2^ on surfaces and achieving a similar reduction of 3.5 Log CFU/mL against planktonic cells. In turn, Qiao et al. (2022) effectively deactivated 12- and 48-h-old *Streptococcus mutans* biofilms using PAW, demonstrating a 7–8 Log reduction following treatment.

In a study by Y. ‐M. Zhao et al. (2020), it was found that planktonic Gram-negative bacteria (*Escherichia coli*, *Aeromonas hydrophila*, *Pseudomonas fluorescens*, and *Shewanella putrefaciens*) were more susceptible to PAW than Gram-positive bacteria (*Listeria innocua* and *Staphylococcus aureus*). Similarly, Miranda et al. (2023) reported that PAW was equally effective against both *E. coli* and *S. aureus* planktonic cells. Additionally, Hozák et al. (2018) tested PAW against both planktonic and biofilm forms of *Staphylococcus epidermidis* and *E. coli*, finding that PAW exhibited greater activity against Gram-negative bacteria. In our study, *E. coli* and *S. aureus*, in both planktonic and biofilm states, followed a similar inactivation pattern, demonstrating that the PAW produced in this study was effective against both bacterial species, regardless of differences in cell wall structure. However, planktonic *E. coli* cells were more susceptible to PAW after 5 and 10 min of treatment with PAW produced at 1000 Hz, as well as after 10 min of treatment with PAW generated at 1500 Hz. Otherwise, no significant differences (p < 0.05) were observed between the treatment groups of both pathogens. PAW physicochemical characterisation revealed that activation under the current conditions resulted in the production of significant concentrations of H_2_O_2_, NO_2_^−^ and NO_3_^−^. It has been suggested that the primary mechanism responsible for bacterial inactivation by PAW is the generation of RONS in high concentrations during plasma treatment. RONS, including short lived hydroxyl radicals (•OH) and nitric oxide (NO) radicals, induce oxidative stress in microbial cells, ultimately leading to the damage of critical cellular components, including membranes, proteins, DNA, and RNA, and resulting in cell death (Zhou et al. 2020; Mai-Prochnow et al. 2021). Moreover, RONS and protons can accumulate inside bacterial cells via pore transport mechanisms, leading to intracellular RONS accumulation and a decrease in cytoplasmic pH. This acidification causes extensive damage to proteins, lipids, DNA, and carbohydrates, further contributing to bacterial inactivation (Wang et al. 2023). Additionally, the synergistic effect of acidic pH and high ORP enhances the antibacterial efficacy of PAW. The combined impact of oxidative stress, intracellular acidification, and membrane disruption ultimately leads to cell rupture, metabolic dysfunction, and bacterial death (Zhou et al. 2020; Zhao et al. 2020; Mai-Prochnow et al. 2021; Wang et al. 2023; Wong et al. 2023).

However, in our study, the produced PAW had a mildly acidic pH (~ 6), suggesting that intracellular acidification was not the dominant mechanism of inactivation. Instead, oxidative stress caused by the long-lived RONS detected in our PAW, such as hydrogen peroxide, nitrite and nitrate, likely played a more critical role. This supports the notion that RONS contribute more significantly to the decontamination activity of PAW than acidity alone.One of the factors that can considerably interfere with PAW or any other decontaminant, suppressing its antimicrobial action, is organic matter contamination (Yemmireddy and Hung 2015; Teng et al. 2018; Yokoyama et al. 2021; Şahiner et al. 2022). In food production facilities, organic residues such as food debris, proteins, and fats are commonly found on processing equipment, food contact surfaces, and work environments (Durek et al. 2019). To assess the impact of organic matter on PAW's antimicrobial effectiveness, its inactivation activity was tested against both planktonic cells and biofilms in the presence of four concentrations of organic matter (0, 0.001, 0.01, and 0.1 g/L BSA).

Both *E. coli* and *S. aureus*, in planktonic and biofilm states, exhibited a similar pattern of decreased viable counts after 20 min of exposure to PAW. In the absence of organic matter, bacteria levels dropped below the detection limit (1 log CFU/ml). However, the antimicrobial activity of PAW decreased with increasing BSA concentrations. It is noteworthy that even the lowest BSA concentration (0.001 g/L) caused viable cell counts to rise significantly, confirming the negative effect of organic load on PAW's antimicrobial activity.

Xiang et al. (2019) reported a dramatic reduction in PAW’s disinfection effectiveness against *E. coli* and *S. aureus* in the presence of peptone or beef extract. In their study, PAW was produced using an atmospheric pressure plasma jet (APPJ) system and sterile distilled water. They suggested that the presence of organic load altered the physicochemical properties of PAW, including pH, oxidation–reduction potential, and reactive species levels, ultimately leading to reduced antimicrobial activity. As the concentration of organic matter increased, ORP and nitrite levels decreased, while pH values increased, demonstrating the inhibitory effect of organic matter on PAW’s disinfection potential. This reduction in efficacy may result from the direct interaction between organic compounds and reactive species, which neutralizes these agents and diminishes their antimicrobial potential, along with alterations in pH and ORP. In turn, Baek et al. (2020) tested PAW produced using a PBR and sterile deionized water for its efficacy against *S. Typhimurium* with varying concentrations of organic matter (0, 0.005, 0.05, 0.1, and 0.5 g/L). Their results demonstrated that bactericidal action decreased in a dose-dependent manner, with higher organic matter concentrations inhibiting the effects of plasma-bubble treatment.

To further evaluate the properties of stored PAW, we assessed its antimicrobial efficacy against biofilms of both pathogens (in the presence or not of organic matter), as biofilms are more commonly encountered in real-world environments compared to planktonic cells, making these results more relevant for practical applications. Consistent with findings from previous studies (Shen et al. 2016; Wang et al. 2023), our results indicate that prolonged storage can negatively affect PAW’s antimicrobial activity, even when stored at 4 °C. PAW stored for 24 h retained comparable efficacy to freshly produced PAW, however, a marked decline in activity was observed after 48 h of storage. This decrease in efficacy aligns with the observed reduction in the concentrations of H₂O₂, NO₂⁻ and NO₃⁻. The observed reductions are most likely linked to oxidative reactions (Shen et al. 2016) and chemical capture of nitrate ions by divalent cations (Mg^2^⁺ and Ca^2^⁺) naturally occurring in tap water used to produce the PAW (Lee et al. 2021).

By contrast, a recent study by (Agus et al. (2025) showed that PAW stored for 72 h at 25 °C retained its antimicrobial properties, despite a reduction in NO₂⁻ and H₂O₂ concentrations and stable levels of NO₃⁻. These findings suggest that the interplay between various RONS may compensate for individual losses, depending on storage conditions and initial PAW composition. Interestingly, the pH of PAW in our study remained relatively stable, likely due to the buffering capacity of ions normally present in tap water and the slower degradation of acidic species under refrigeration. H₂O₂ also showed only a slight, but significant decline, which may have helped retain partial antimicrobial activity even after 48 h of storage. Similar results were reported in a study by Shen et al. (2016), where the pH remained relatively stable during storage at 4 °C despite a decline in key RONS concentrations. Furthermore, we also tested the antimicrobial activity of stored PAW against *E. coli* and *S. aureus* biofilms in the presence of organic matter. As expected, the presence of organic load in the form of 0.01 g/L BSA further reduced PAW’s decontamination potential, confirming its susceptibility to interference by organic load.

While this study demonstrated significant inactivation of pathogens by PAW, it is important to note that CFU enumeration may not fully reflect the total number of viable bacterial cells. Previous research by (Sun et al. 2024) showed that PAW treatment can induce a viable but nonculturable state in bacteria. Therefore, complementary methods such as fluorescence microscopy or viability qPCR should be considered in future work to more accurately assess bacterial survival. Importantly, PAW exposure was followed by rapid dilution in PBS to reduce residual activity; however, no additional neutralization steps were applied. This may represent a limitation, as residual reactive species could potentially affect diluted samples. Future studies should include a neutralisation step to eliminate this variable and ensure accurate post-treatment enumeration. Lastly, PAW was generated using a single batch of sterilised tap water; however, tap water composition varies by source and may affect PAW properties. Future studies should evaluate water variability and develop standardisation strategies for broader industrial application.

Since the application of PAW may involve direct contact with human skin, testing its toxicity on skin cells can ensure its safety for human exposure, minimise risks of irritation or damage and support regulatory approval. This evaluation will help balance efficacy and biocompatibility, ensuring PAW is both effective and safe for practical applications. In this study, PAW’s cytotoxicity was tested on human keratinocytes to assess its safety for use. We demonstrated that PAW did not exhibit a cytotoxic effect even after 30 min of exposure. Although HaCaT cell viability declined following a longer exposure period of 45 min, the value remained above the normative cell viability threshold (70%) (Cannella et al. 2019). Similarly, Lee et al. (2022) reported that PAW, produced using deionized water and a microwave plasma system, had no adverse effects on fibroblast and HaCaT cells, as neither cell death nor proliferation was induced. In a study by Nastasa et al. (2021), the toxicity of PAW generated using a GlidArc-based reactor was assessed after long-term exposure. Mice were given PAW as drinking water for 90 days, and no toxic effects were observed following the treatment. Sampaio et al. (2022) reported that the in vitro exposure of oral keratinocytes to distilled and deionized PAW, generated using a gliding arc plasma jet, for 20 min resulted in increased cell viability, with no cytotoxic effects observed even after prolonged exposure for 60 min.

## Conclusion

This study demonstrated PAW’s strong antibacterial efficacy against both *E. coli* O157 and *S. aureus*, effectively inactivating planktonic and biofilm cells and achieving significant reductions even at short treatment times and reductions below the detection limit with longer treatments. The results show that higher discharge frequency during production enhances PAW’s antimicrobial effectiveness. Also, the use of atmospheric air along with tap water to produce PAW streamlines production and large-scale implementation.

However, the study also highlights a key challenge: PAW’s antimicrobial activity is significantly reduced in the presence of even very low amounts of organic contaminants. This shows the importance of pre-cleaning strategies before PAW application to maximize its disinfection potential, as well as the need to enhance PAW’s antimicrobial efficiency through combination treatments, optimized activation parameters, or real-time monitoring systems that account for organic load. Importantly, we also demonstrated that PAW retains antimicrobial activity after 24 h of refrigerated storage, although its effectiveness declined after 48 h, particularly in the presence of organic matter.

Furthermore, PAW demonstrated an excellent safety profile, with minimal cytotoxic effects on human keratinocytes even after prolonged exposure. Given its strong efficacy and low cytotoxicity, PAW may be particularly well-suited for surface disinfection in agri-food environments, either as a standalone treatment in low-soiling conditions or as part of integrated hygiene protocols in environments where organic contamination is present. Findings in this study support PAW’s viability as a safe and effective alternative to conventional chemical disinfectants.

## Clinical trial number

Not applicable

## Competing Interests

The authors declare no competing interests.

## Data Availability

The data that support the findings of this study are not openly available due to reasons of sensitivity and are available from the corresponding author upon reasonable request.

## References

[CR1] Agus R, Pipoz L, Avino F, Lavrikova A, Myers B, Furno I (2025) Plasma-activated water retains antimicrobial properties against *Escherichia coli* after 72 h of storage. Plasma Phys Control Fusion 67:015014. 10.1088/1361-6587/ad9950

[CR2] Ansari A, Parmar K, Shah M (2022) A comprehensive study on decontamination of food-borne microorganisms by cold plasma. Food Chem Mol Sci 4:100098. 10.1016/j.fochms.2022.100098 10.1016/j.fochms.2022.100098 PMC9235041 35769398

[CR3] Baek KH, Heo YS, Park JY, Kang T, Lee YE, Lim J, Kim SB, Jo C (2020) Inactivation of *Salmonella Typhimurium* by non-thermal plasma bubbles: exploring the key reactive species and the influence of organic matter. Foods 9:1689. 10.3390/foods9111689 33218136 10.3390/foods9111689 PMC7698966

[CR4] Beutin L, Martin A (2012) Outbreak of Shiga toxin–producing *Escherichia coli* (STEC) O104:H4 infection in Germany causes a paradigm shift with regard to human pathogenicity of STEC strains. J Food Prot 75:408–418. 10.4315/0362-028X.JFP-11-452 22289607 10.4315/0362-028X.JFP-11-452

[CR5] Bridier A, Briandet R, Thomas V, Dubois-Brissonnet F (2011) Resistance of bacterial biofilms to disinfectants: a review. Biofouling 27:1017–1032. 10.1080/08927014.2011.626899 22011093 10.1080/08927014.2011.626899

[CR6] Cannella V, Altomare R, Chiaramonte G, Di Bella S, Mira F, Russotto L, Pisano P, Guercio A (2019) Cytotoxicity evaluation of endodontic pins on L929 cell line. BioMed Res Int 2019:3469525. 10.1155/2019/3469525 31815131 10.1155/2019/3469525 PMC6877943

[CR7] Durek J, Schlüter O, Fröhling A (2019) Utilising cold plasma for equipment cleaning and disinfection. Ref Modul Food Sci. 10.1016/B978-0-08-100596-5.21371-1

[CR8] Ercoli L, Gallina S, Nia Y, Auvray F, Primavilla S, Guidi F, Pierucci B, Graziotti C, Decastelli L, Scuota S (2017) Investigation of a staphylococcal food poisoning outbreak from a Chantilly cream dessert, in Umbria (Italy). Foodborne Pathog Dis 14:407–413. 10.1089/fpd.2016.2267 28402712 10.1089/fpd.2016.2267 PMC5512467

[CR9] Fernandes S, Gomes IB, Simões M, Simões LC (2024) Novel chemical-based approaches for biofilm cleaning and disinfection. Curr Opin Food Sci 55:101124. 10.1016/j.cofs.2024.101124

[CR10] Gao Y, Francis K, Zhang X (2022) Review on formation of cold plasma activated water (PAW) and the applications in food and agriculture. Food Res Int 157:111246. 10.1016/j.foodres.2022.111246 35761559 10.1016/j.foodres.2022.111246

[CR11] Gott RP, Engeling KW, Olson J, Franco C (2023) Plasma activated water: a study of gas type, electrode material, and power supply selection and the impact on the final frontier. Phys Chem Chem Phys 25:5130–5145. 10.1039/D2CP03489A 36722991 10.1039/d2cp03489a

[CR12] Hadinoto K, Astorga JB, Masood H, Zhou R, Alam D, Cullen PJ, Prescott S, Trujillo FJ (2021) Efficacy optimization of plasma-activated water for food sanitization through two reactor design configurations. Innov Food Sci Emerg Technol 74:102867. 10.1016/j.ifset.2021.102867

[CR13] Hadinoto K, Niemira BA, Trujillo FJ (2023) A review on plasma-activated water and its application in the meat industry. Compr Rev Food Sci Food Saf 22:4993–5019. 10.1111/1541-4337.13250 37799092 10.1111/1541-4337.13250

[CR14] Han Q-Y, Wen X, Gao J-Y, Zhong C-S, Ni Y-Y (2023) Application of plasma-activated water in the food industry: a review of recent research developments. Food Chem 405:134797. 10.1016/j.foodchem.2022.134797 36371834 10.1016/j.foodchem.2022.134797

[CR15] Hozák P, Scholtz V, Khun J, Mertová D, Vaňková E, Julák J (2018) Further contribution to the chemistry of plasma-activated water: influence on bacteria in planktonic and biofilm forms. Plasma Phys Rep 44:799–804. 10.1134/S1063780X18090040

[CR16] Kemper MJ (2012) Outbreak of hemolytic uremic syndrome caused by *E. coli* O104:H4 in Germany: a pediatric perspective. Pediatr Nephrol 27:161–164. 10.1007/s00467-011-2067-7 22160440 10.1007/s00467-011-2067-7

[CR17] Le HHT, Dalsgaard A, Andersen PS, Nguyen HM, Ta YT, Nguyen TT (2021) Large-scale *Staphylococcus aureus* foodborne disease poisoning outbreak among primary school children. Microbiol Res (Pavia) 12:43–52. 10.3390/microbiolres12010005

[CR18] Lee GJ, Lamichhane P, Ahn SJ, Kim SH, Yewale MA, Choong CE, Jang M, Choi EH (2021) Nitrate capture investigation in plasma-activated water and its antifungal effect on *Cryptococcus pseudolongus* cells. Int J Mol Sci 22(23):12773. 10.3390/ijms222312773 34884579 10.3390/ijms222312773 PMC8657772

[CR19] Lee HR, Lee YS, You YS, Huh JY, Kim K, Hong YC, Kim C-H (2022) Antimicrobial effects of microwave plasma-activated water with skin protective effect for novel disinfectants in pandemic era. Sci Rep 12:5968. 10.1038/s41598-022-10009-1 35396389 10.1038/s41598-022-10009-1 PMC8992786

[CR20] Lianou A, Panagou EZ, Nychas G-JE (2017) Meat safety—I foodborne pathogens and other biological issues. In: Toldrá F (ed) Lawrie’s meat science. Elsevier, Amsterdam, pp 521–552

[CR21] Liu C, Chen C, Jiang A, Sun X, Guan Q, Hu W (2020) Effects of plasma-activated water on microbial growth and storage quality of fresh-cut apple. Innov Food Sci Emerg Technol 59:102256. 10.1016/j.ifset.2019.102256

[CR22] Mai-Prochnow A, Zhou R, Zhang T, Ostrikov K, Mugunthan S, Rice SA, Cullen PJ (2021) Interactions of plasma-activated water with biofilms: inactivation, dispersal effects and mechanisms of action. NPJ Biofilms Microbiomes 7:11. 10.1038/s41522-020-00180-6 33504802 10.1038/s41522-020-00180-6 PMC7841176

[CR23] Marches A, Clement E, Albérola G, Rols M-P, Cousty S, Simon M, Merbahi N (2022) Cold atmospheric plasma jet treatment improves human keratinocyte migration and wound closure capacity without causing cellular oxidative stress. Int J Mol Sci 23:10650. 10.3390/ijms231810650 36142561 10.3390/ijms231810650 PMC9504313

[CR24] Mendoza IC, Luna EO, Pozo MD, Vásquez MV, Montoya DC, Moran GC, Romero LG, Yépez X, Salazar R, Romero-Peña M, León JC (2022) Conventional and non-conventional disinfection methods to prevent microbial contamination in minimally processed fruits and vegetables. LWT 165:113714. 10.1016/j.lwt.2022.113714 10.1016/j.lwt.2022.113714 PMC9239846 35783661

[CR25] Měřínská T, Walker M, Keener K (2025) Using plasma-activated water for decontamination of *Salmonella* spp. on common building surfaces in poultry houses. Food Microbiol 126:104673. 10.1016/j.fm.2024.104673 39638442 10.1016/j.fm.2024.104673

[CR26] Miranda FS, Tavares VKF, Gomes MP, Neto NFA, Chiappim W, Petraconi G, Pessoa RS, Koga-Ito CY (2023) Physicochemical characteristics and antimicrobial efficacy of plasma-activated water produced by an air-operated coaxial dielectric barrier discharge plasma. Water (Basel) 15:4045. 10.3390/w15234045

[CR27] Moonsub K, Seesuriyachan P, Boonyawan D, Rachtanapun P, Sawangrat C, Opassuwan T, Wattanutchariya W (2024) Combating foodborne pathogens: efficacy of plasma-activated water with supplementary methods for *Staphylococcus aureus* eradication on chicken, and beef. Food Chem X 24:101890. 10.1016/j.fochx.2024.101890 39498257 10.1016/j.fochx.2024.101890 PMC11533612

[CR28] Nastasa V, Pasca A-S, Malancus R-N, Bostanaru A-C, Ailincai L-I, Ursu E-L, Vasiliu A-L, Minea B, Hnatiuc E, Mares M (2021) Toxicity assessment of long-term exposure to non-thermal plasma activated water in mice. Int J Mol Sci 22:11534. 10.3390/ijms222111534 34768973 10.3390/ijms222111534 PMC8583710

[CR29] Oliveira M, Fernández-Gómez P, Álvarez-Ordóñez A, Prieto M, López M (2022) Plasma-activated water: a cutting-edge technology driving innovation in the food industry. Food Res Int 156:111368. 10.1016/j.foodres.2022.111368 35650984 10.1016/j.foodres.2022.111368

[CR30] Qian J, Zhuang H, Nasiru MM, Muhammad U, Zhang J, Yan W (2019) Action of plasma-activated lactic acid on the inactivation of inoculated *Salmonella Enteritidis* and quality of beef. Innov Food Sci Emerg Technol 57:102196. 10.1016/j.ifset.2019.102196

[CR31] Qiao D, Li Y, Pan J, Zhang J, Tian Y, Wang K (2022) Effect of plasma activated water in caries prevention: the caries related biofilm inhibition effects and mechanisms. Plasma Chem Plasma Process 42:801–814. 10.1007/s11090-022-10244-4

[CR32] Rahman M, Hasan MdS, Islam R, Rana R, Sayem A, Sad MdAA, Matin A, Raposo A, Zandonadi RP, Han H, Ariza-Montes A, Vega-Muñoz A, Sunny AR (2022) Plasma-activated water for food safety and quality: a review of recent developments. Int J Environ Res Public Health 19:6630. 10.3390/ijerph19116630 35682216 10.3390/ijerph19116630 PMC9180626

[CR33] Rothwell JG, Alam D, Carter DA, Soltani B, McConchie R, Zhou R, Cullen PJ, Mai-Prochnow A (2022) The antimicrobial efficacy of plasma-activated water against *Listeria* and *E. coli* is modulated by reactor design and water composition. J Appl Microbiol 132:2490–2500. 10.1111/jam.15429 34957649 10.1111/jam.15429

[CR34] Rothwell JG, Hong J, Morrison SJ, Vyas HKN, Xia B, Mai-Prochnow A, McConchie R, Phan-Thien K-Y, Cullen PJ, Carter DA (2023) An effective sanitizer for fresh produce production: in situ plasma-activated water treatment inactivates pathogenic bacteria and maintains the quality of cucurbit fruit. Microbiol Spectr. 10.1128/spectrum.00034-23 37428084 10.1128/spectrum.00034-23 PMC10434273

[CR35] Şahiner A, Halat E, Yapar EA, Kara BA (2022) Evaluation of organic load related efficacy changes in antiseptic solutions used in hospitals. Turk J Med Sci 52:825–833. 10.55730/1300-0144.5379 36326304 10.55730/1300-0144.5379 PMC10390165

[CR36] Sampaio AdaG, Chiappim W, Milhan NVM, Botan Neto B, Pessoa R, Koga-Ito CY (2022) Effect of the pH on the antibacterial potential and cytotoxicity of different plasma-activated liquids. Int J Mol Sci 23:13893. 10.3390/ijms232213893 36430372 10.3390/ijms232213893 PMC9693261

[CR37] Shen J, Tian Y, Li Y, Ma R, Zhang Q, Zhang J, Fang J (2016) Bactericidal effects against *S. aureus* and physicochemical properties of plasma-activated water stored at different temperatures. Sci Rep 6:28505. 10.1038/srep28505 27346695 10.1038/srep28505 PMC4921907

[CR38] Sun Y, Gao R, Liao X, Shen M, Chen X, Feng J, Ding T (2024) Stress response of *Salmonella Newport* with various sequence types toward plasma-activated water: viable but nonculturable state formation and outer membrane vesicle production. Curr Res Food Sci 8:100764. 10.1016/j.crfs.2024.100764 38779345 10.1016/j.crfs.2024.100764 PMC11109322

[CR39] Tan J, Karwe MV (2021) Inactivation and removal of *Enterobacter aerogenes* biofilm in a model piping system using plasma-activated water (PAW). Innov Food Sci Emerg Technol 69:102664. 10.1016/j.ifset.2021.102664

[CR40] Teng Z, Luo Y, Alborzi S, Zhou B, Chen L, Zhang J, Zhang B, Millner P, Wang Q (2018) Investigation on chlorine-based sanitization under stabilized conditions in the presence of organic load. Int J Food Microbiol 266:150–157. 10.1016/j.ijfoodmicro.2017.11.027 29216555 10.1016/j.ijfoodmicro.2017.11.027

[CR41] Thirumdas R, Kothakota A, Annapure U, Siliveru K, Blundell R, Gatt R, Valdramidis VP (2018) Plasma activated water (PAW): chemistry, physico-chemical properties, applications in food and agriculture. Trends Food Sci Technol 77:21–31. 10.1016/j.tifs.2018.05.007

[CR42] Tropea A (2022) Microbial contamination and public health: an overview. Int J Environ Res Public Health 19:7441. 10.3390/ijerph19127441 35742689 10.3390/ijerph19127441 PMC9224327

[CR43] U.S. Food and Drug Administration (2023) Foodborne pathogens. https://www.fda.gov/food/outbreaks-foodborne-illness/foodborne-pathogens. Accessed 27 Jan 2025

[CR44] U.S. Food and Drug Administration (2024) Outbreak investigation of Escherichia coli O157:H7: Onions (October 2024). https://www.fda.gov/food/outbreaks-foodborne-illness/outbreak-investigation-e-coli-o157h7-onions-october-2024. Accessed 24 Jul 2025

[CR45] UK Health Security Agency (2024) Investigation into an outbreak of Shiga toxin-producing E. coli (STEC) O145 in Great Britain, May to June 2024. https://www.gov.uk/government/publications/shiga-toxin-producing-e-coli-outbreak-o145-may-to-june-2024/investigation-into-an-outbreak-of-shiga-toxin-producing-e-coli-stec-o145-in-great-britain-may-to-june-2024. Accessed 27 Jul 2025

[CR46] Vyas HKN, Xia B, Alam D, Gracie NP, Rothwell JG, Rice SA, Carter D, Cullen PJ, Mai-Prochnow A (2023) Plasma activated water as a pre-treatment strategy in the context of biofilm-infected chronic wounds. Biofilm 6:100154. 10.1016/j.bioflm.2023.100154 37771391 10.1016/j.bioflm.2023.100154 PMC10522953

[CR47] Wang H, Han R, Yuan M, Li Y, Yu Z, Cullen PJ, Du Q, Yang Y, Wang J (2023) Evaluation of plasma-activated water: Efficacy, stability, physicochemical properties, and mechanism of inactivation against *Escherichia coli*. LWT 184:114969. 10.1016/j.lwt.2023.114969

[CR48] Wong KS, Chew NSL, Low M, Tan MK (2023) Plasma-activated water: physicochemical properties, generation techniques, and applications. Processes 11:2213. 10.3390/pr11072213

[CR49] World Health Organization (2025) Food safety. https://www.who.int/news-room/fact-sheets/detail/food-safety. Accessed 6 Jan 2025

[CR50] Xiang Q, Kang C, Zhao D, Niu L, Liu X, Bai Y (2019) Influence of organic matters on the inactivation efficacy of plasma-activated water against *E. coli* O157:H7 and *S. aureus*. Food Control 99:28–33. 10.1016/j.foodcont.2018.12.019

[CR51] Yemmireddy VK, Hung Y-C (2015) Effect of food processing organic matter on photocatalytic bactericidal activity of titanium dioxide (TiO₂). Int J Food Microbiol 204:75–80. 10.1016/j.ijfoodmicro.2015.03.019 25863338 10.1016/j.ijfoodmicro.2015.03.019

[CR52] Yokoyama T, Miyazaki S, Akagi H, Ikawa S, Kitano K (2021) Kinetics of bacterial inactivation by peroxynitric acid in the presence of organic contaminants. Appl Environ Microbiol 87:e01860–20. 10.1128/AEM.01860-20 33127816 10.1128/AEM.01860-20 PMC7783340

[CR53] Zhao Y-M, Ojha S, Burgess CM, Sun D-W, Tiwari BK (2020) Inactivation efficacy and mechanisms of plasma-activated water on bacteria in planktonic state. J Appl Microbiol 129:1248–1260. 10.1111/jam.14677 32358824 10.1111/jam.14677

[CR54] Zhao Y, Bhavya ML, Patange A, Sun D, Tiwari BK (2023a) Plasma-activated liquids for mitigating biofilms on food and food contact surfaces. Compr Rev Food Sci Food Saf 22:1654–1685. 10.1111/1541-4337.13126 36861750 10.1111/1541-4337.13126

[CR55] Zhao Y, Zhang Z, Ning Y, Miao P, Li Z, Wang H (2023b) Simultaneous quantitative analysis of *Escherichia coli*, *Staphylococcus aureus* and *Salmonella typhimurium* using surface-enhanced raman spectroscopy coupled with partial least squares regression and artificial neural networks. Spectrochim Acta A Mol Biomol Spectrosc 293:122510. 10.1016/j.saa.2023.122510 36812753 10.1016/j.saa.2023.122510

[CR56] Zheng X, Liang S, Wen L, Wang H, Bai Y, Wang Y, Cao L, Li D, Guo J, Chen Y, Wang C, Liu X, Zhang C (2025) Foodborne outbreak of enterotoxigenic *Staphylococcus aureus* at a school in Hebei, China. Food Res Int 214:116645. 10.1016/j.foodres.2025.116645 40467232 10.1016/j.foodres.2025.116645

[CR57] Zhou R, Zhou R, Wang P, Xian Y, Mai-Prochnow A, Lu X, Cullen PJ, Ostrikov K, Bazaka K (2020) Plasma-activated water: generation, origin of reactive species and biological applications. J Phys D Appl Phys 53:303001. 10.1088/1361-6463/ab81cf

